# Early and late onset complications of gynaecologic surgery: a multimodality imaging approach

**Published:** 2017-03

**Authors:** I De Blasis, V Vinci, ME Sergi, F Capozza, M Saldari, F Moro, MC Moruzzi, AC Testa, L Manganaro

**Affiliations:** Catholic University of the Sacred Heart, Obstetrics and Gynaecology, Rome; University of Rome Sapienza, Department of Radiological Sciences

**Keywords:** Gynaecologic surgery, postoperative complications, ultrasound, Computed Tomography (CT), Magnetic Resonance Imaging

## Abstract

The role of imaging after surgery is pivotal to drive clinical management of early and/or late onset complications. Most frequently used imaging technique after pelvic surgery is Ultrasound (US), Magnetic Resonance Imaging (MRI) and Computed Tomography (CT). While Ultrasound is a standard procedure, using grey scale and/or colour Doppler evaluation, MRI and CT scan protocols should be derived on the basis of the specific indication of the exam. Correct evaluation of female pelvis after gynaecologic surgery, having in mind the most frequent complications, is based on the correct use of the instruments and on the experience of the examiner, who should be aware of the history of the patient, type of surgery and clinical symptoms for which the exam is required; the clinician should be aware of the possibilities and limits of the different techniques, in order to choose the most appropriate imaging modality and promptly make a correct diagnosis.

## Introduction

The role of radiological imaging after surgery is pivotal to drive clinical management of early and late onset complications. For correct evaluation and diagnosis of possible complications, is important for feature’s interpretation to know preoperative conditions, type of surgical procedures, histological diagnosis and the “new anatomy” of the pelvis. In this article we wish to describe radiological findings of the most frequent postoperative complications after gynaecological surgery, according to specific pathologies and distinguish between early and late onset complications.

## Benign Preoperative Pathologies

Benign preoperative pathologies may be distinguished in delayable and urgent conditions.

Among the first category, those that more frequently require to plan an elective surgical intervention are represented by endometriosis, benign ovarian cysts and uterine myomas.

– Endometriosis, the presence of ectopic endometrial glands and tissue, is a common gynaecologic condition, occurring in 6%–10% of the general female population (Bulletti et al., 2010). Ectopic endometrial glands can develop into the ovaries as endometriotic cysts, also called endometriomas, into the myometrium as adenomyosis, or spread in the peritoneum as superficial or deep endometriosis (Exacoustos et al., 2014). Even if it is a benign condition, surgical treatment can be very extended and varies on the basis of localization, sometimes requiring a bowel resection.

– Benign ovarian cysts represent a wide category that include different histotype, where the most common are endometriomas, serous cystadenomas and teratomas (Testa et al., 2014). Those lesions are usually easy to identify with Ultrasound, and often are asymptomatic and diagnosed as incidental findings. Correct surgical treatment of these lesions in young women is represented by cystic enucleation or, if not possible, unilateral adnexectomy. 

– Myomas are the most common benign tumours of the genital organs in women of childbearing age, and can be single, but are more often multiple, causing significant morbidity, and deterioration of quality of life (Sparic et al., 2016). Surgical treatment of uterine myomas depends on the number of myomas, the localization and the age of the patient, and can consist in single or multiple myomectomy or in total/subtotal hysterectomy. Urgent benign gynaecological conditions are mainly represented by haemorrhagic corpus luteum, extra uterine pregnancy, and adnexal torsion.

– Corpus luteum cyst-wall rupture is a rare complication that occurs most frequently in women in their reproductive age (Fiaschetti et al., 2014). When bleeding occurs, haemorrhage may spread into the peritoneal cavity causing hemoperitoneum. Corpus luteum haemorrhage can be managed with a conservative approach, with the aim of preserving ovarian function as well as eliminate the source of bleeding.

– Ectopic pregnancy affects 1–2% of all pregnant women (Barnhart, 2009). Most ectopic pregnancies are located in the Fallopian tube, and when indicated surgical treatment can vary between salpingectomy (radical treatment) or salpingotomy (conservative treatment) (Hajenius et al., 2007).

– Torsion of the ovary, tube, or both is responsible for approximately 3% of all gynaecologic emergencies (Hibbard, 1985) and is responsible for acute or subacute congestion and ischemia of the ovary. Often ovarian torsion is associated with the presence of ovarian cysts that can enlarge the volume of the ovary increasing the risk of its torsion (Albayram and Hamper, 2001). Treatment depends on the state of ovarian parenchyma, which if mostly necrotic may require ovariectomy.

## Malignant Pathology

The incidence of female genital tract malignancy varies on the type of tumour, and when the tumour is resectable, surgery represents the treatment of choice.

– Cervical cancer is the second most common malignancy in women worldwide (Boyle et al., 2003). Treatment depends mainly on tumour FIGO (International Federation of Gynecology and Obstetrics) stage. In early disease, minimally invasive surgery is possible, while women with advanced disease will require neoadjuvant treatment (e.g. radiotherapy or chemotherapy alone or combined), possibly followed by surgery (Testa et al., 2014).

– Endometrial cancer is the most common gynaecological malignancy in industrialised countries (Boyle et al., 2003). Incidence differs between rural and urban populations and across countries, indicating that lifestyle has an effect, in particular obese women are at high risk to develop endometrial cancer (Purdie et al., 2001; Calle and Kaaks, 2004).

– Ovarian cancer is the most aggressive gynaecological malignancy. The five-year survival rate of patients is around 40% and the disease accounts for approximately half of all deaths related to gynaecological cancer (Siegel et al., 2014; Berrino et al., 2007). The most important factor for survival is stage at diagnosis (Howlader et al., 2014). At the time of diagnosis, more than 75% of patients have an advanced stage of disease, and their five-year survival rate is very poor (Jelovac and Armstrong, 2011). Although most patients with advanced ovarian cancer respond well to standard management—primary cytoreductive surgery followed by platinum/taxane-based adjuvant chemotherapy—the majority of those patients develop recurrent disease and die of progressive disease.

– Uterine sarcomas account for approximately 1% of all female genital tract malignancies and 3%−7% of all uterine cancers (Major et al., 1993). Tumour stage is the most important prognostic factor, and the behaviour of this tumour is very aggressive. Leiomyosarcomas are associated with poor prognosis even when confined to the uterus at the time of diagnosis (Abeler et al., 2009; D’Angelo et al., 2009). Recurrence rate ranges from 53% to 71% (Mayerhofer et al., 1999; Koivisto-Korander et al., 2008).

## Surgery

Surgical procedures are modulated on the type of gynaecological pathology (benign or malignant lesions), extension of the disease, age of the patient (fertility-sparing surgery):

– Cervical conisation and trachelectomy, indicated in case of pre-neoplastic lesions (i.e. CIN 3) or early stage cervical cancer in young patients. The most frequent early complication is minor bleeding, in particular when receiving cold knife conisation (Santesso et al., 2016).

– Ovarian cyst enucleation and/or unilateral adnexectomy, indicated in cases of benign ovarian cysts or borderline ovarian tumours in young patients. The incidence of injury to internal organs is rare, but patients with prior surgeries, history of pelvic infection, endometriosis, or other causes of adhesive disease are at greater risk. Ureteral injury may occur, and a common location of ureteral injury is at the pelvic brim, where it is in close proximity to the infundibulopelvic ligament (Thompson, 1997). Other rare complications are bleeding from the infudibulopelvic ligament and bowel damage (in cases of significant adhesions).

– Total hysterectomy (with abdominal or laparoscopic approach), with complete resection of the uterus, indicated in case of benign pathologies or endometrial cancer, when confined to the uterus; this procedure can be associated with bilateral salpingectomy or bilateral salpingoophorectomy (depending on the age of the patient) and pelvic and/or paraortic lymphadenectomy. In these cases will residuate only the vaginal vault. A common early complication of this technique is the vault haematoma, while urinary tract and bowel injuries and peri-operative bleeding are the most severe injuries occurring during hysterectomy. Ureter and bladder complications have a predominance in laparoscopic hysterectomy (Mäkinen et al., 2001).

– Radical hysterectomy, with complete resection of the uterus and parametrium; indicated in case of cervical cancer, often following neoadjuvant treatment and following Querleu-Monroe classification, can be divided into four types of radicality (A–D) based on lateral extent of resection (Querleu and Morrow, 2008). This surgical procedure is generally associated with bilateral salpingoophorectomy and pelvic and/or paraortic lymphadenectomy. Most frequent postoperative complications after radical hysterectomy are urinary complications (cystocele, stress or postural incontinence, vescicovaginal fistula, ureterovaginal fistula), urinary tract infection, pelvic lymphocyst (or lymphocele), deep venous thrombosis, hematoma, ileus (Yin and Giu, 2016).

– Debulking surgery, that consists in total hysterectomy, bilateral salpingoophorectomy, omentectomy, pelvic and/or paraortic lymphadenectomy, appendectomy and excision of all the visible implants of carcinosis (sometimes requiring bowel resection). This is a very extended and demolitive surgical procedure, indicated in case of advanced ovarian cancer, eventually following neoadjuvant treatment. Because of the aggressive type of surgery, it is connected to several complications, mostly represented by bleeding and bowel injuries, occurring around in 10% of cases (Venesmaa and Ylikorkala, 1992).

## Postoperative imaging techniques

Most frequently used imaging techniques after pelvic surgery are Ultrasound (US), both transabdominal and transvaginal approaches, Magnetic Resonance Imaging (MRI) and Computed Tomography (CT) scan. While Ultrasound is a standard procedure, using grey scale and/or colour Doppler evaluation, MRI and CT scan protocols should be conducted on the basis of the specific indication of the exam. Ultrasound is a commonly available, non-invasive, and inexpensive imaging method that can be carried out without any risk for the patient. Ultrasound also has the advantage of being a dynamic and interactive examination that can provide information on how pelvic and abdominal structures move in relation to each other (Fischerova and Burgetova, 2014). A high-end ultrasound machine with sensitive Doppler, and equipped with high-resolution (5.0–9.0 MHz) endocavitary and transabdominal convex and linear array probes, is needed. For the diagnosis of possible postoperative complications, transabdominal scan of the abdomen and pelvis can allow an immediate panoramic evaluation. Transvaginal sonography (TVS) is the most widely available technique to evaluate the gynaecological internal organs, but also adjacent pelvic structures (e.g. bladder, rectum, sigmoid colon, pelvic lymph nodes, and peritoneum) (Fischerova, 2011). However, if it can be considered the gold standard in the preoperative evaluation of uterine and adnexal pathology it does not represent the first choice technique in the postoperative setting; indeed, the transvaginal approach is not recommended immediately after surgery, in case of hysterectomy, because of the risk of rupture of the vaginal wound and an experienced sonographer or gynaecologist is required due to the difficulties in the diagnosis of haemorrhage.

**Figure 1:**
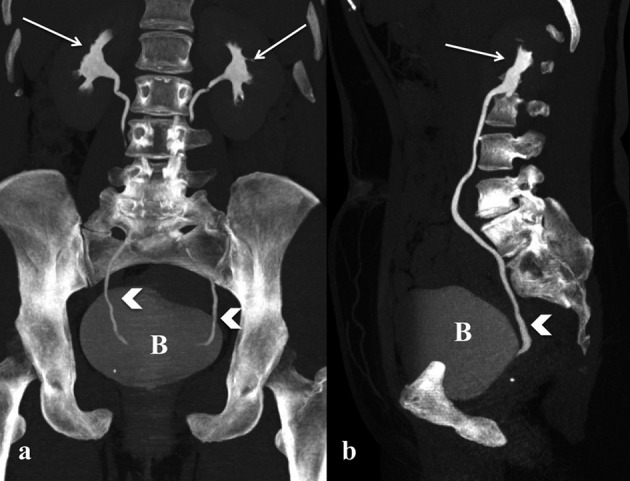
— a-b: MIP reconstruction obtained in coronal and sagittal plane. CT Urography shows the iodinate urine in the renal pelvis (white arrow), ureter (white arrowhead) and bladder (B).

A limitation of transabdominal Ultrasound can be a poor quality image because of intestinal bloating (common condition after surgery), elevated BMI or discomfort of the patient because of the recent surgical wound in case of early postoperative evaluation. When US findings are equivocal or the extent of findings are beyond the field-of-view or obscured by bowel gas, CT scan represents the imaging modality of choice, especially indicated in the acute abdomen and pelvis (Cano Alonso et al., 2009; Yitta S et al., 2011). In fact CT is easily available in all institutes, is well tolerated by most of the patients even in critical conditions, it is rapid and offers a complete evaluation of the abdomen and pelvis allowing adjunctive information compared to standard US, regarding for example active bleeding, renal function, bowel perforation and others. The standard protocol of CT scan should include abdominal and pelvis evaluation; if not indicated otherwise, intravenous (IV) administration of contrast should be performed with acquisition before and after IV injection in arterial phase and portal venous phase (respectively 30-40 sec and 70-80 sec scan delay after contrast injection). When urinary tract injury is suspected, it is mandatory to implement the standard protocol with an acquisition in the excretory phase, a delayed scan at 7-12min after contrast injection in order to obtain the opacification of the renal pelvis, ureters and bladder (CT Urography) (Fig. 1). In order to evaluate small bladder injury, a CT Cystography may be performed, which consists of an administration through urinary catheter of an enema solution (50mL in 500mL normal saline) with scan acquisition before and after, having previously clamped catheter. Furthermore, when a bowel injury is suspected, oral contrast should be administered 20 minutes before the scan protocol. Once the protocol is acquired, images should be reconstructed at 3mm with multiplanar reconstruction software (MPR) on coronal and sagittal plane or using Maximum Intensity Projection software (MIP). The MIP algorithm is particularly useful to obtain angiography and urography-like images.

At last, an important role is covered by MRI, which offers a valid alternative to multidetector CT (MDCT) especially in selected cases and followup although few disadvantages such as great cost, limited availability, and generally lengthy examination. MRI, thanks to its multiparametric and multiplanar yield, offers a higher contrast resolution on soft tissue, which finds its best application in cases of fistula. Standard MRI exam should always include a previous IV administration of anti-peristaltic agent, and should be performed on a high magnet field (> 1T magnet), with patient preferably on supine position and using pelvic phased-array multichannel coil. Standard protocol of the pelvis, performed in our Institute, usually include: axial, sagittal and coronal fast spin-echo (TSE) T2 weightd (W) sequences; axial spin-echo (SE) T1-W sequence; axial Fat Saturated T1-W and T2-W; Diffusion weighted sequences (DWI) with b value of 0-500 and 1000; axial and sagittal multiphase contrast enhanced 3D T1-W sequences after administration of intravenous gadolinium. 

## Postoperative physiological findings

As previously introduced, “new anatomy” of the pelvis depends on the type of surgical procedures. In case of conservative surgery on the cervix, as conisation or trachelectomy, will residuate corpus uteri and ovaries, while in case of conservative surgery on the ovaries, will residuate ovarian parenchima, that can appear smaller than the contralateral and with a little scar or calcification on the surface where the cyst has been enucleated. In case of total or radical hysterectomy, uterus and ovaries will be absent, and will residuate the vaginal vault, that can appear very thin or thick, in particular on the angles, and depending on patient’s healing, can simulate a small recurrent lesion. In particular, on transvaginal US the exploration of the pelvis includes the visualization of the vaginal vault, bladder, rectum, mesorectum, and pelvic lateral walls until visualization of iliac vessels and Psoas muscle. In case of extended citoreduction for ovarian cancer, some additional surgical trace could be present, as bowel resection, omentectomy, appendectomy, splenectomy. All surgical traces should be signed on the report.

## Risk factors for postoperative complications after gynaecological surgery

Some preoperative risk factors are associated with increased postoperative complications after gynaecologic surgery, in particular age ≥ 80 years old, medical comorbidities, dependent functional status, and unintentional weight loss (Erekson et al., 2011). Rate and type of postoperative complications depends on type of surgery, increasing with the complexity of surgical procedures. In particular peri operative complications are more frequent after surgery for gynaecological malignancies, because some invasive procedures like lymphadenectomy are routinely performed. Type of hysterectomy has also an impact on postoperative complications, more frequent in radical hysterectomy than in total hysterectomy (Yin and Giu, 2016). Moreover, neoadjuvant treatment, in particular chemoradiation, can influence the rate of postoperative complications as reported in a recent study by Ferrandina et al. (2014). Debulking surgery for advanced ovarian cancer is a type of surgery that is extended to the upper abdomen, often including bowel and diaphragmatic resection, therefore postoperative complications are not strictly confined to the pelvis or urinary tract, but are similar to those that can follow general surgery.

## Imaging findings of postoperative complications

Postoperative complications can be divided in early and late onset, defined as any adverse event occurring within or after 30 days from surgery (Table I).

**Table I:** List of possible postoperative complications and preferred imaging technique for the evaluation.

TYPE OF COMPLICATION	US	CT	MRI
– Early onset complication			
Hemorrhage and hematoma		++	+
Urinary tract injuries	+	++	+
Fluid collection and infection	++		
Bowel injuries		++	
Complications of caesarean section	+	+	+
Lymphocele	++	+	
Thrombosis		++	+
– Late onset complication			
Urinary tract complications	+	++	+
Bowel injuries		++	
Fistula		+	++

+ = suitable; ++ = more suitable; empty box= not indicated, US = Ultrasound, MRI = Magnetic Resonance Imaging, CT = Computed Tomography

## Early onset complications

**Figure 2:**
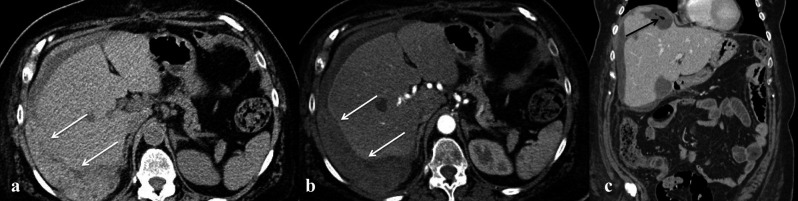
— Patient with serous ovarian carcinoma who underwent resection of carcinomatosis and hepatic nodules metastasectomy. a) non contrast axial CT image which shows an inhomogeneous hyperdense collection next to liver (due to blood content; white arrows); b) axial contrast-enhanced CT images showing no active extravasation; c) coronal contrast CT image which shows the extent of the collection in the subdiaphragmatic space and the presence of gas bubbles (black arrows) within the collection on coronal suggestive for infection.

**Figure 3:**
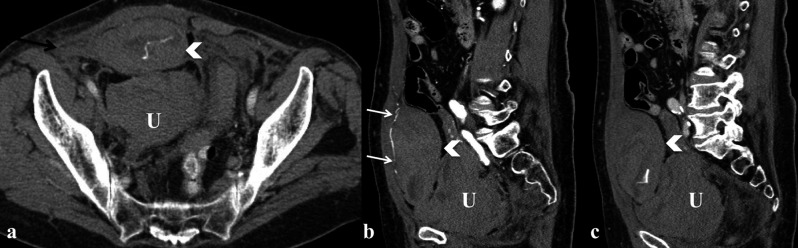
— Patient who underwent hysterectomy for fibromatosis of the uterus (U). Post-contrast images oriented on axial (a) and sagittal (b, c) planes, which show an hematoma (white arrowhead) within the right rectum of the abdomen supplied by a branch of the epigastric artery (dense linear striation, white arrows) ; the hematoma is in communication with an intraperitoneal blood collection (black arrow).

**Figure 4:**
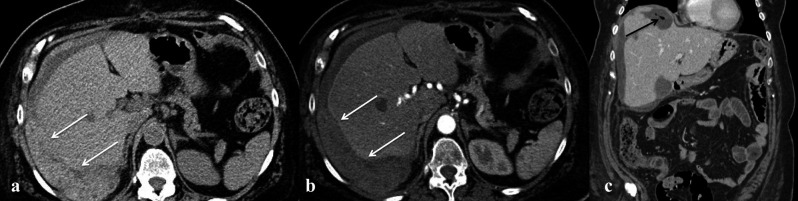
— Delayed post contrast CT images oriented on axial (a), coronal (b) and sagittal (c) planes, showing hydroureteronephrosis (white arrowhead) with ipsilateral urinoma (white arrows) due to postsurgery adhesions.

**Figure 5 a-b:**
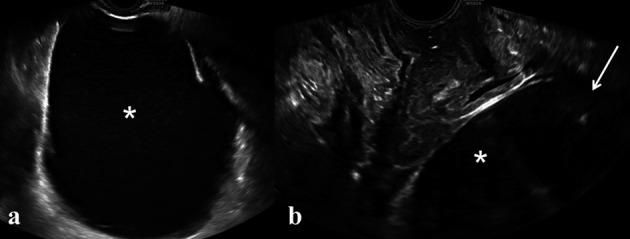
— Grey scale image of pelvic fluid collection. On transvaginal examination it appears as a cystic ovoidal lesion (a, white asterisk), localized upon the vaginal vault, with low level content (b, white arrow).

**Figure 6:**
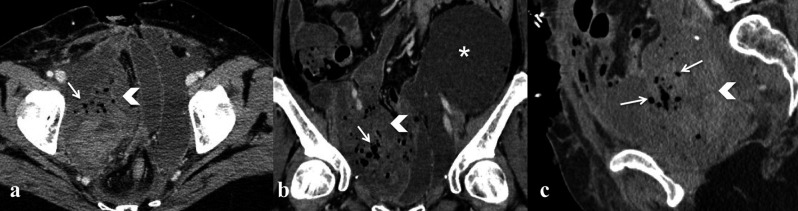
— Pelvic collection (a-b; white arrowhead) with inhomogeneous content and enhancing, thickened wall; presence of gas bubbles (a-c, white arrows) and a huge left-sided lymphocele (b, white asterisk).

**Figure 7:**
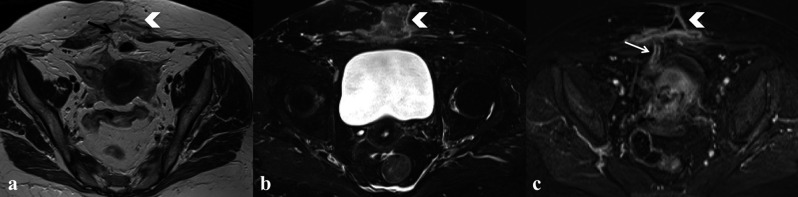
— Patient who had undergone right oophorectomy for a pelvic inflammatory disease. a) T2 WI on axial plane which shows a subcutaneous abscess (white arrowhead) with strands towards the uterus and dehiscence of the surgical wound (black arrow); b) T2 WI with fat suppression on axial plane showing subcutaneous abscess (white arrowhead); c) T1 contrast WI on axial plane which show enhancement of the subcutaneous tissue and a fistula (white arrow).

**Figure 8:**
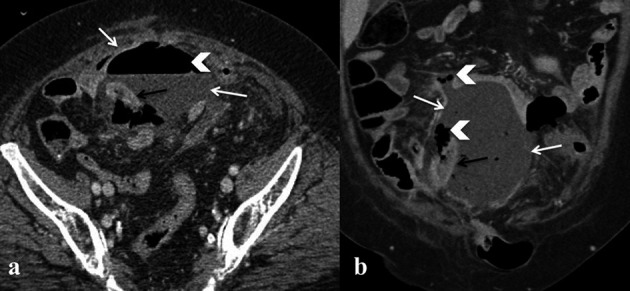
— Bowel injury in a Patient who had undergone chemotherapy and cytoreductive surgery. a-b) axial and coronal Contrast-enhanced CT images show a massive abscess (white arrow) containing air (white arrowhead) and enteric material surrounding a small bowel loop (black arrow).

**Figure 9:**
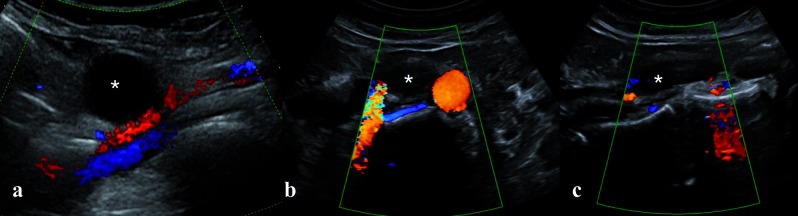
— Color Doppler images of pelvic (a) and lomboaortic (b, c) lymphoceles (white asterisks) after systematic lymphadenectomy for cervical cancer. On ultrasound it appears as a unilocular lesion with anechoic content and no signal on color Doppler examination.

– Haemorrhage and hematoma represent the most common complications after gynaecological surgery. On transabdominal US, it is possible to distinguish between a suprafascial hematoma (within the abdominal wall) and intraperitoneal hematoma. However, in the suspicion of this type of complication, CT scan represents the imaging of choice, because in the early phase the hematoma will result in a higher attenuation area (70-90 UH) in unenhanced CT (Fig.2), while an haemorrhage will be seen as an active extravasation of medium contrast either in the arterial phase or in the venous one, when of minor entity (Takeda et al., 2008) (Fig. 3). Extensive haemorrhage may require intervention either by interventional radiology or by open surgery. A particular type of hematoma is the vault hematoma, frequent during the first week after radical hysterectomy and often self-healing.

– Urinary tract injuries have an incidence of 1% of women who undergo pelvic surgery and includes different type of complications, either with an early onset or late onset. Up to 75% of ureteric injuries are caused by gynaecologic surgery, and most of these occur in case of radical hysterectomy and lymph node dissection (Bai et al., 2006). Bladder lesion are the most frequent and usually are resolved intraoperatively (Takeda et al., 2008); ureteral transection represents an infrequent and early complication with consequent uro-peritoneum or urinomas (Fig. 4). These complications can be related to a direct accidental surgical damage or can result after ischemic trauma. Injury is suspected postoperatively with continued vaginal drainage, peritonitis, flank pain, or rising creatinine. Ureteral tract lesions are not usually evaluable with US, but in case of a low tract ureteral lesion with the development of a urinoma, this can be identified with transvaginal ultrasound examination as unilocular lesion with anechoic content, and with colour Doppler intralesional pulsatic signal (Testa et al., 2009). On the contrary, ureteral lesions may appear on CT scan as a leakage of iodinate urine within the abdomen observed on a delayed acquisition phase at 7-10 minutes after contrast administration (excretory phase) with a sided urinoma. Uro-peritoneum is seen on US and CT as free fluid in the abdomen.

– Fluid collection and infection. First imaging modality to evaluate the presence of fluid collection or abscess is represented by US (Fig. 5); in case of simple fluid collection, it will appear as anechoic content, while in case of abscess the content will appear more dense and corpusculated; as an advantage of contrast-enhanced CT scan, it will not only consent the diagnosis but often allows to evaluate the cause. In fact infection and abscess are usually due to coexisting visceral injury. Infection of a fluid collection or lymphocele, as well as abscess, should be suggested on CT scan in case of collection with inhomogeneous content and enhancing and thickened wall; the presence of gas bubbles is highly suspicious for infection of gram-negative bacteria (Fig. 6). A further possible complication of infection can be represented by fistulisation of the abscess with surrounding tissues, in this case MRI will offer a detailed image (Fig. 7).

– Bowel injury. As well as urinary tract injury, these complications may appear early or late after surgery. The early type complications include bowel perforation, which in urgent condition may be confirmed on a plain radiography of the abdomen, showing free air in the subdiaphragmatic space. Further investigation is represented by CT scan which confirm the persistence of free air within the abdomen, and allow to directly visualize the site of the perforation, usually associated with the adjacent presence of fluid collection and small gas bubbles within it (Fig.8); in few cases it is possible to highlight a small perforation with an oral contrast administration of gadolinium followed by an unenhanced CT scan 20 min after assumption, in this case it could be seen an extravasion of oral contrast from the bowel lumen into the peritoneum.

– Complications after caesarean section. Normal appearance consists of a focal linear hypoattenuation which should not be confused with uterine dehiscence, which is a rare complication (around 0.6 – 3.8%). A small collection of fluid may be easily seen at the site of the caesarean section with US. In case of uterine dehiscence and subsequent infection, CT images demonstrate a defect within the uterine segment incision in direct communication with a fluid/gas collection. In addiction, rare complications after caesarean section can be also represented by hematoma or haemorrhage.

– Lymphocele formation is a complication that occurs following lymphadenectomy. Reports of the incidence of asymptomatic lymphoceles following oncogynaecological procedures involving lymphadenectomy range from 1% to 58% (Ferguson and Maclure, 1961). A symptomatic lymphocele may compress adjacent structures (ureters, urinary bladder, rectum or large vessels) and consequently cause pain, hydronephrosis, urinary urgency or thrombosis (Zikan et al., 2015). The most serious complication of lymphocele is infection (Achouri et al., 2013). On Ultrasound, lymphocele usually appears as a uni- or multilocular, round or oval cystic structure with a thick wall and fluid content of varying echogenicity, which might contain thin septae (Zikan et al., 2015). Usually it is not vascularized at colour doppler examination (Fig. 9). In case of doubt relating to the nature of the lesion, in case of poor quality ultrasound image (i.e. due to elevated BMI or intestinal bloating) or in case of clinical suspicion of complications related to the size or localization of the lesion, CT scan should be performed. On CT scan it will appear as a multiloculated hypoattenuating fluid mass with absent of week capsular enhancement, often localized along the lymphatic drainage; in case of complication such as infection it may appear with a higher attenuating content which may include also gas bubbles, thickened and enhancing wall, and a reactive response of the adjacent fat tissue.

– Thrombosis: Ovarian vein thrombosis is encountered as an early postpartum complication, however it may also occur in case of abdominopelvic surgery for malignancies such as debulking surgery for ovarian cancer. It is usually depicted on MDCT images as an hypoattenuating filling defect within the ovarian vein eventually associated to surrounding soft tissue stranding in case of inflammation (thrombophlebitis) (Mantha et al., 2015). In doubt cases, excretory phase may help to distinguish between a non-opacified vein and the ureters. Its recognition is important because it may be complicated by thrombosis of the inferior vena cava and in rare cases by pulmonary thromboembolism.

## Late onset complications

**Figure 10:**
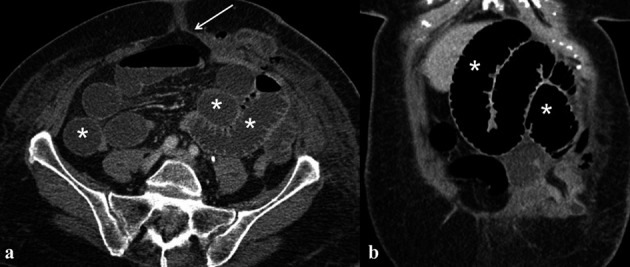
— a-b: Bowel obstruction in a Patient with previous laparoscopic resection of ovarian endometrioma. a-b) axial and coronal CT images show air-fluid level in dilatated small bowel loops (white asterisks) with erniation through the surgical access (white arrow).

**Figure 11:**
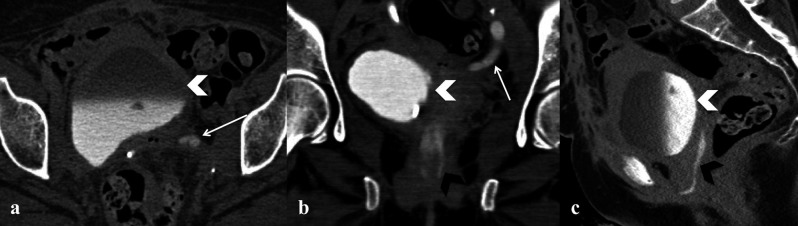
— Uretero-vaginal fistula in Patient who had undergone surgery for cervical cancer. Axial (a), coronal (b) and sagittal (c) delayed CT images show iodinate urine within the bladder (white arrowhead) and communication between the ureter and the vagina with contrast in it (black arrowhead) and associated left ureteronephrosis (white arrow).

– Urinary tract complications: as written above, urinary tract complication may either appear on early or chronic phase. The late urinary tract complications include ureteral stenosis ending in hydronephrosis, size discrepancy between the kidneys with a reduction of the affected one; both these aspects may be studied on common transabdominal US. However, CT scan with contrast injection allows to evaluate the renal function which, if impaired, will be seen as a delayed enhancement of the kidney on arterial phase with a delayed or absent excretory phase compared to the other normal kidney.

– Bowel injury. In the late onset, bowel injuries are included bowel obstruction mostly due to adhesions. Adhesions are the most common cause of bowel obstruction after surgery, and they constitute the leading cause of long-term re-intervention following abdominal and pelvic surgery; these can lead to complete obstruction, intermittent or lowgrade bowel obstruction. The diagnosis of smallbowel obstruction due to adhesions is presumed when all other causes of obstruction have been ruled out on CT scan; main findings include a narrow zone of transition without an identifiable lesion, acute angulation of the small-bowel loops and traction deformities (Hongo et al., 2011; Sandrasegaran et al., 2005) (Fig. 10). In urgent condition bowel obstruction may be suspected also on plain abdominal radiography, seen as dilatated bowel loop containing air-fluid levels.

– Fistula: it is a delayed complication, often due to a bowel or urinary injury; it occurs mostly in case of malignant indication to surgery and previous radiotherapy. The imaging of choice is MRI thanks to its higher spatial resolution compared to CT, for example it offers an excellent sensibility and specificity (100% and 86%) with regard to perianal fistula (Villa et al., 2012). The fistulous tract can be optimally visualized with standard T2 weighted images with fat saturation as an hyperintense fluid filled tract, often associated to inflammatory reactive change of the adjacent adipose tissue seen as hyperintense strands within it; in case of active inflammation fistula can also be visualized on post contrast images as hypointense tract with enhancing wall. After pelvic surgery, most of fistulous connections are vesicovaginal and rectovaginal, however these may also be enterocutaneous , enterovescical, enterovaginal etc. In case of suspected vescico-vaginal or ureteral- vagina fistula, CT urography may be helpful thanks to the direct evaluation of the fistulous tract filled with mdc in the late excretory phase (Fig. 11). On the suspect of fistula between bowel (intestinal loop or rectum) and the vagina, it could be useful to administer oral contrast either orally or by endorectal catheter.

## Conclusion

Correct evaluation of female pelvis after gynaecologic surgery, having in mind the “new anatomy” and the most frequent complications following surgery, is based on the correct use of the instruments and on the experience of the examiner, who should be aware of the history of the patient, the type of surgical treatment and the clinical symptoms for which the exam is required. On the other hand, in this wide panorama of different imaging modalities and specific protocols, the clinician should be aware of the possibilities and limits of the different techniques, in order to choose the most appropriate imaging modality and promptly make a correct diagnosis. When the patient is clinically stable and compliant, Ultrasound should be considered for the first approach for the evaluation of lymphocele, ascites, fluid collection and hydronephrosis. In case of worsening of clinical conditions, acute abdomen or when active bleeding is suspected, MDTC is the examen of choice thanks to its fast acquisition and multiphasic modality. MRI finds an important role in the assessment of late onset complications, such as fistulas, thanks to its high contrast resolution. In this setting a multidisciplinary approach is essential to improve the correct management of these patients.
